# A Framework for Cancer Surveillance in Japan

**DOI:** 10.2188/jea.15.199

**Published:** 2005-11-07

**Authors:** Satoshi Kaneko

**Affiliations:** 1 Statistics and Cancer Control Division, Research Center for Cancer Prevention and Screening, National Cancer Center.

**Keywords:** Neoplasms, Epidemiology/prevention & control, Registries, Japan

## Abstract

The World Health Organization (WHO) has recommended that countries develop national cancer control programs in order to reduce the number of deaths due to preventable cancers. The national cancer control program should be comprehensive and systematic with evidence-based priority-setting and the efficient use of limited resources. In order to provide evidence-based information, cancer surveillance systems must be established with registration as a focus. Cancer registration monitors the incidence, mortality, survival, and prevalence of cancers. In Japan, however, cancer registration systems have not been either well developed or standardized until recently. In 2003, the Ministry of Health, Labour and Welfare of Japan launched the Third Term Comprehensive 10-Year Strategy Program for Cancer Control, which gave grants to several projects to enhance the dissemination and standardization of cancer registries. However, the establishment of a cancer registration system is merely the first step in the process to provide a comprehensive surveillance system that leads to a national cancer control program, as proposed by the WHO. To provide the best cancer care services equitably in Japan, cancer surveillance systems should be established without delay.

## Introduction

The World Health Organization (WHO) has recommended to member states that they develop national cancer control programs in order to reduce the number of deaths due to preventable cancers, and to improve the quality of life among cancer patients and their families. This resolution was adopted at the 58th World Health Assembly held in Geneva, on May 25, 2005. The WHO cancer control program is based on a comprehensive and systematic approach using evidence-based priority setting and efficient use of limited resources. The first step to such an approach is to develop a cancer surveillance system to track the cancer burden in a nation. This includes the monitoring of incidence, mortality, person-years of life lost (PYLLs), survival, and prevalence of cancers.^1^ After the data are collected, the following steps need to be taken: (1) the detection of impediments to cancer control, including epidemiological studies on cancer, (2) studies for possible and feasible strategies to implement effective cancer control programs against the impediments, (3) implementation of the strategies, and (4) evaluation of the strategies using the surveillance data.^2^ Thus, cancer control strategy is a continuous and recurring process.

Japan has not had a national cancer control strategy that is recognized as such by public health and health policy management. Because of this lack of strategy, cancer registration systems, which are the main source of cancer surveillance data, have not been well developed nor standardized until today. In 2003, however, the Ministry of Health, Labour and Welfare launched the Third Term Comprehensive 10-Year Strategy Program for Cancer Control, which gave grants to several projects to enhance the dissemination and standardization of cancer registries. The following two projects are the most important ones: a project to standardize and improve the quality of prefecture-wide cancer registries (principle investigator: T. Sobue, National Cancer Center), and a project to disseminate hospital-based cancer registries in the ministry-designated regional cancer treatment hospitals (principle investigator: H. Ikeda, National Cancer Center). In this article, I introduce the two projects and discuss their progress as well as describe the features of a future cancer surveillance system in Japan.

## Current Cancer Surveillance Systems in Japan

In July 2004, the project for “the standardization and improvement of prefecture-wide cancer registries in Japan” conducted a baseline survey on the current status of cancer registration with collaboration of the Japanese Association of Cancer Registries. As a result, 34 prefectures of the all of 47 ones have been running cancer registries, but their Death Certificate Notified (DCN) rates, which are a surrogate index for the completeness of cancer registration, do not come up to the standards of the “developed countries of cancer registration.” Furthermore, the registration items and definitions are not consistent among registries, which shows that standardization of prefecture-wide cancer registries is an urgent matter for the establishment of cancer surveillance systems in Japan. The reporting from hospitals needs to be improved as well.^3^

The development of a national surveillance system, called a hospital-based cancer registry, for the monitoring and evaluation of cancer care among hospitals, has been delayed. Most cancer treatment hospitals in Japan have not installed a facility-based cancer registration system. The available data for cancer care are limited to clinical data that are managed by physicians. Even if a hospital has a facility-based cancer registration system, the registry items and definitions are defined independently, not standardized nationally.

## Developing a Standardized Cancer Registration System in Japan under the Third Term Comprehensive 10-Year Strategy Program for Cancer Control

### 1. Quality improvement and standardization of prefecture-wide cancer registries

The project established eight goals and standards for prefecture-wide cancer registries (Table). The project selected 15 high-quality cancer registries in order to support the introduction of a standardized database system developed by the Radiation Effects Research Foundation, Hiroshima. Furthermore, several working groups under the project have promulgated rules and information required for standardization; e.g., terminology, the rule for multiple primary cancers, the reporting format from facilities and the structure of databases, and the effective usage of national statistics data. The conclusion of the working groups will be opened to the public at the Web site: http://ncrp.ncc.go.jp (in Japanese). The project succeeded cancer registry data collection for national cancer incidence estimations, which had been conducted by The Research Group for Population-Based Cancer Registries in Japan from 1975 through 1999,^4^ from 2000 cancer incidence estimation.

**Table.** Eight items of standards and objectives to be achieved during the next 10 years in prefecture-wide cancer registries in Japan.

(1) Legislative Authority	The official approval process for cancer registration must be completed.
(2) Data Content and Format	Cancer retistry data must be contain standard 25 items, that includs 12 items required by the National Cancer Center for incidence monitoring data at the national level
(3) Data Completeness	Cancer registry must fulfill the standard of completeness of registration: I/M (incidece/mortality) ratio, Death Certificate Notification (DCN, %), and Death Certificate Only (DCO, %) . The value of the standard to be announced.
(4) Data Timeliness	Cancer registry must fulfill the standard of timeliness of registration; The incidence data must be available within defined period. The value of the standard period to be announced.
(5) Data Quality	Cancer registry must operate an error monitoring system.
(6) Follow-up	Cancer registy must follow-up the registered cases.
(7) Annual Reporting	Cancer registry must issue annual reports.
(8) Data Use	Cancer registry data can be used for for research purposes.

### 2. Dissemination of a standardized hospital-based cancer registry in the ministry-designated regional cancer treatment hospitals

Since 2002, the Ministry of Health, Labour and Welfare of the Japanese government has sponsored the ministry-designated regional cancer treatment hospital program, in which the ministry designates a hospital in every second level medical region (363 regions) in all prefectures in order to ensure high-quality care for every cancer patient regardless of his or her place of residence. A hospital willing to be designated needs to fulfill all the requirements for the designation. One of the requirements is to have hospital-based cancer registry management. However, most of hospitals in Japan have not installed a facility-based cancer registration system; thus, dissemination of standardized hospital-based cancer registries, especially for the ministry-designated regional cancer treatment hospitals, is an urgent matter for the cancer surveillance system. Standardized registration terms and their definitions were fixed in 2003 by the previous project team. They were authorized by the Ministry of Health, Labour and Welfare and disseminated to the ministry-designated regional cancer treatment hospitals through prefectural governments. In order to disseminate and support standardized registration in hospitals, the project has also developed registration software (obtainable from the Web site at http://jcdb.ncc.go.jp) and provides training programs. The project has been tackling several tasks, e.g., updating the standards in order to be consistent with the prefecture-wide cancer registry standards, running training programs for cancer registry personnel, and preparing to start a certified cancer registrar approval program in Japan.

## Future Cancer Surveillance Systems: from Cancer Surveillance to a National Cancer Control Program

Cancer surveillance systems are regular data-collecting systems that gather data from primary to tertiary prevention care for cancer. They regularly keep track of unusual or problematic events that are related to cancer in a population. Although they do not solve the problems by themselves, they are necessary for guiding the next steps, e.g., epidemiologic studies and further data collection. Among the surveillance systems, cancer registries are a mainstay because calculation of incidences is the first step to tracking cancer burden in a nation or region. In recent decades, the establishment of cancer registration systems has been popular especially among developed countries. In the United States, most of states have established high-quality cancer registration systems with support through the National Cancer Institute and the Centers for Disease Control and Prevention (CDC).^5^^,^^6^ Following the establishment of the state cancer registration systems, cancer health policy of the US has shifted to the construction of a national framework in which the surveillance systems are connected.^7^ Using this framework, the US is planning to manage the national comprehensive cancer control programs effectively and efficiently.^8^

How about the cancer surveillance systems in Japan? In the future, similar to the US, all the surveillance systems should be connected to each other. In Japan, however, cancer surveillance systems that regularly collect data from primary to tertiary cancer care are not well developed. Therefore, the establishment of a standardized cancer registration system and its effective use of registry data should have priority over the establishment of other surveillance systems for cancer. Fortunately, the Third Term Comprehensive 10-Year Strategy Program for Cancer Control has started and the National Cancer Center has taken initiatives on the cancer surveillance functions (Figure). Simultaneous with the cancer registry standardization, pattern of care studies should be planned. The pattern of care studies are the hypothesis-testing studies focused on specific cancer sites and stages that compare results regarding survival rates, quality of life, and other measurements among prefectures and hospitals. The results from the pattern of care studies will be used for standardization of cancer prevention activities in a region or cancer care services in a hospital, leading to high-quality cancer care with equity for all individuals in each region and hospital. Furthermore, establishment and networking of other cancer surveillance systems are needed. A behavioral risk factor surveillance system, a cancer screening data collecting system, a recurrence and metastasis detection program, and an end-of-life care surveillance system will give us indicators about cancer control activities and cancer care if their data are mutually connected and effectively used.

**Figure.**
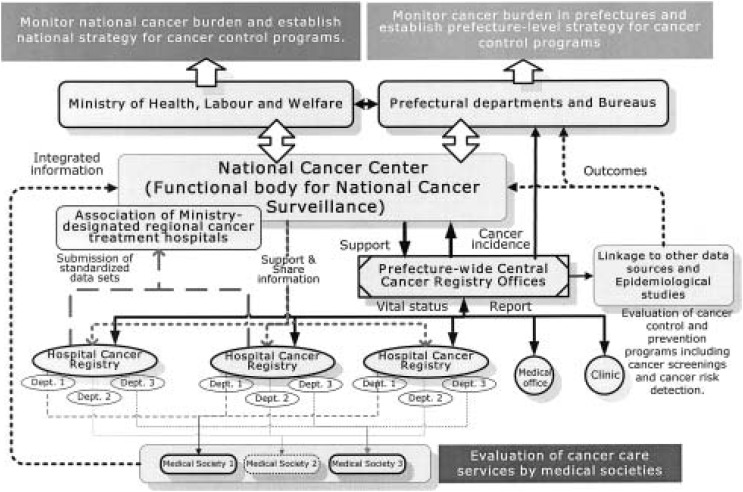
Framework of a national cancer surveillance system in Japan.

To provide the highest quality and the most equitable cancer care and services for all individuals in Japan, the establishment and networking of cancer surveillance systems covering primary to tertiary cancer care are needed. All individuals, parties, and organizations related to cancer care and services in Japan should collaborate and make efforts to establish the framework for effective cancer surveillance systems.
